# Gender differences in anticholinergic overactive bladder medication use and the risk of dementia

**DOI:** 10.1002/alz.094590

**Published:** 2025-01-09

**Authors:** Kathryn Richardson, Yoon Loke, Nick Steel, Duncan Edwards, Jalesh N Panicker, Stuart Irving, Katharina Mattishent, Chris Fox, Louise Robinson, Irene Petersen

**Affiliations:** ^1^ University of East Anglia, Norwich United Kingdom; ^2^ University of Cambridge, Cambridge, Cambridgeshire United Kingdom; ^3^ University College London, London United Kingdom; ^4^ University of Exeter, Exeter United Kingdom; ^5^ Newcastle University, Newcastle upon Tyne United Kingdom

## Abstract

**Background:**

Studies that report an association between anticholinergic medications and dementia often suffer from confounding by indication and rarely consider gender effects. We estimated the association between recurrent prescriptions for anticholinergic overactive bladder (OAB) medications and incident dementia, separately in men and women.

**Method:**

We studied patients aged ≥50 years first prescribed an anticholinergic OAB drug (e.g. oxybutynin, solifenacin, tolterodine) during 1998‐2019 in England using the Clinical Practice Research Datalink Aurum (CPRD) linked to hospital admissions data. To reduce confounding by indication, we compared patients receiving a second prescription to those only prescribed one during the first year. To reduce protopathic bias, we began follow‐up for incident dementia (first diagnosis in CPRD, hospital data or prescription for a cognitive enhancer) first prescription, and censored patients at death, leaving the practice, one month before last data collection, or 31/3/2020. We excluded patients with history of dementia, cognitive impairment, serious central nervous system disorders, severe mental illness, alcohol abuse, or <12 months registration with their GP. We used Cox regression, with age as the time‐scale, to estimate hazard ratios (HR) for recurrent OAB prescription and incident dementia adjusted for many socio‐demographic and health‐related covariates, comorbidities, and concomitant medications, separately in men and women. We also examined risks by drug and cumulative defined daily doses (DDDs).

**Result:**

We included 80,874 men and 149,930 women who initiated bladder anticholinergics with ≥3 years of subsequent follow‐up. Of these, 83,718 (received only one prescription during the first year. Receiving a second prescription was associated with a greater increased dementia incidence in men (HR = 1.23, 95%CI 1.17‐1.29) than women (HR = 1.11, 95%CI 1.08‐1.15; p = 0.002 for gender interaction). Associations increased with greater cumulative exposure, with HRs (95%CI) of 1.32 (1.24‐1.40) and 1.16 (1.11‐1.21) for >6 months of DDDs (vs ≤1 month) for men and women.

**Conclusion:**

We observed greater increased rates of dementia for men with recurrent OAB anticholinergic prescriptions than women, and associations were dose‐dependent. Prescribers should exercise additional caution in men, and aim for alternative management options or the lowest effective dose and a limited period where possible.

